# Kidney Ischemia/Reperfusion Injury Induces Changes in the Drug Transporter Expression at the Blood–Brain Barrier *in vivo* and *in vitro*

**DOI:** 10.3389/fphys.2020.569881

**Published:** 2020-11-12

**Authors:** Malgorzata Burek, Sandra Burmester, Ellaine Salvador, Kerstin Möller-Ehrlich, Reinhard Schneider, Norbert Roewer, Michiaki Nagai, Carola Y. Förster

**Affiliations:** ^1^Department of Anaesthesia and Critical Care, University of Würzburg, Würzburg, Germany; ^2^Division of Nephrology, Department of Medicine I, University of Würzburg, Würzburg, Germany; ^3^Department of Cardiology, Hiroshima City Asa Hospital, Hiroshima, Japan

**Keywords:** kidney ischemia/reperfusion injury, brain pathology, blood–brain barrier, drug transporter, tight junctions

## Abstract

Ischemia/reperfusion injury is a major cause of acute kidney injury (AKI). AKI is characterized by a sudden decrease in kidney function, systemic inflammation, oxidative stress, and dysregulation of the sodium, potassium, and water channels. While AKI leads to uremic encephalopathy, epidemiological studies have shown that AKI is associated with a subsequent risk for developing stroke and dementia. To get more insights into kidney–brain crosstalk, we have created an *in vitro* co-culture model based on human kidney cells of the proximal tubule (HK-2) and brain microvascular endothelial cells (BMEC). The HK-2 cell line was grown to confluence on 6-well plates and exposed to oxygen/glucose deprivation (OGD) for 4 h. Control HK-2 cells were grown under normal conditions. The BMEC cell line cerebED was grown to confluence on transwells with 0.4 μm pores. The transwell filters seeded and grown to confluence with cereEND were inserted into the plates with HK-2 cells with or without OGD treatment. In addition, cerebEND were left untreated or treated with uremic toxins, indole-3-acetic acid (IAA) and indoxyl sulfate (IS). The protein and mRNA expression of selected BBB-typical influx transporters, efflux transporters, cellular receptors, and tight junction proteins was measured in BMECs. To validate this *in vitro* model of kidney–brain interaction, we isolated brain capillaries from mice exposed to bilateral renal ischemia (30 min)/reperfusion injury (24 h) and measured mRNA and protein expression as described above. Both *in vitro* and *in vivo* systems showed similar changes in the expression of drug transporters, cellular receptors, and tight junction proteins. Efflux pumps, in particular Abcb1b, Abcc1, and Abcg2, have shown increased expression in our model. Thus, our *in vitro* co-culture system can be used to study the cellular mechanism of kidney and brain crosstalk in renal ischemia/reperfusion injury.

## Introduction

Decreased kidney function in patients with chronic kidney disease (CKD) or acute kidney injury (AKI) often leads to neurological effects such as cognitive impairment, neuropathy and cerebrovascular disease (Tanaka and Okusa, 2020). Blood-borne toxins, normally eliminated by healthy kidneys, are thought to affect brain function through kidney-brain crosstalk (Lu et al., 2015). In general, in CKD or AKI, the accumulation of uremic toxins in the blood is mainly due to a decreased glomerular filtration rate (GFR). In ischemia/reperfusion injury, reduced GFR is usually due to decreased glomerular perfusion and tubular obstruction due to necrotic and shed tubular epithelial cells. This leads to the accumulation of uremic toxins in the body. Patients with CKD have an increased prevalence of microbleads in the brain, which is associated with an increased risk of stroke and a cognitive decline compared to healthy age-matched controls (Lau et al., 2020). The blood-brain barrier (BBB), an interface between blood and the brain, is the first line of contact with blood-borne toxins (Banks, 2016). Only the toxins, which are able to cross the BBB or modulate cellular processes at the BBB have an influence on neurons in the brain. The BBB is formed by BMECs, which interact with pericytes, astrocytes, microglia and neurons to form a cellular, transporter and metabolic barrier that protects the brain homeostasis (Keaney and Campbell, 2015). Endothelial cells of the BBB are tightly connected by tight and adherens junctions. Transmembrane proteins such as claudins, occludin, junctional adhesion molecules, and VE-cadherin are involved in establishing paracellular permeability (Haseloff et al., 2015). A low level of transendothelial endocytosis and a high expression of efflux transporters contribute to the barrier properties (Krizbai et al., 2016; Wilhelm et al., 2016). In addition, metabolizing enzymes such as cytochrome P450 family limit the amounts of toxins that approach the brain parenchyma (Dauchy et al., 2008). Systemic factors can influence the BMECs and lead to changes in BBB integrity (Lu et al., 2015). In a rat model of a chronic renal failure, a significant decrease of influx and efflux drug transporters on mRNA and protein level have been shown (Naud et al., 2012). Renal failure serum treatment of BMECs *in vitro*, showed similar effects on transporter expression. Interestingly, the brain permeability for drugs remained unchanged suggesting the sustained BBB integrity (Naud et al., 2012). Various uremic toxins are known. One example is indoxyl sulfate (IS), a metabolite of L-tryptophan from food. IS induces oxidative stress and contributes to cardiovascular disease by inhibiting the endothelial proliferation and wound healing (Yu et al., 2011). Organic anion transporters (OAT) at the BBB are involved in the brain uptake and efflux of IS (Ohtsuki et al., 2002). Elevated cerebral IS levels lead to negative effects on neurons (Iwata et al., 2007). Indole-3-acetic acid (IAA), a uremic toxin derived from tryptophan, is highly increased in renal failure and induces apoptosis (Edamatsu et al., 2014). Ischemia/reperfusion injury is a major cause of AKI. We analyze here brain microvessels isolated from mice exposed to bilateral renal ischemia (30 min)/reperfusion (24 h) injury and measured protein and mRNA expression of influx and efflux transporters, cellular receptors and tight junction proteins. We also created an *in vitro* co-culture model based on human proximal tubular kidney cells HK-2 and the BMEC cell line cerebEND. The addition of kidney injury toxins, IAA and IS should mimic serum toxin accumulation (Gondouin et al., 2013). We demonstrate the induction of selected efflux transporters in BMECs and altered tight junction protein expression using this *in vitro* model.

## Materials and Methods

### Reagents

Indoxyl sulfate (IS) and Indole-3-acetic acid (IAA) were purchased from Sigma-Aldrich. A 50 mM stock solution of IAA was made in ethanol and used at a concentration of 1 mM. A 1 M stock solution of IS (potassium salt) was prepared in water and used in a concentration of 1 mM. Solvents alone (1 mM ethanol and 1mM potassium chloride) were used for the control treatment.

### Mouse Brain Tissue and Isolation of Capillaries

Frozen mouse brain tissue from mice exposed to bilateral renal ischemia (30 min)/reperfusion (24 h) injury was provided by Dr. R. Schneider (Division of Nephrology, Department of Medicine I, University of Würzburg). The parameters that characterize the kidney function of sham and clamp-operated mice are shown in Table 1. Brain capillaries were isolated by mechanical separation and centrifugation with 25% bovine serum albumin (Dilling et al., 2017). After washing with phosphate buffered saline (PBS), the capillaries were used for protein and RNA isolation as described below.

**TABLE 1:** Effects of bilateral renal ischemia (30 min)/reperfusion injury (24 h) (clamp) on kidney function in mice.

	Sham	Clamp
Weight (g)	24.6 ± 2.92	25.5 ± 1.69
Urine output volume in 30 min (ml)	0.203 ± 0.11	0.037 ± 0.05*
Inulin-clearance (ml/min/100 g)	1.338 ± 0.43	0.623 ± 0.12*

Data are presented as mean ± SD, *p < 0.05 versus sham group.

### Cell Culture

**FIGURE 1:**
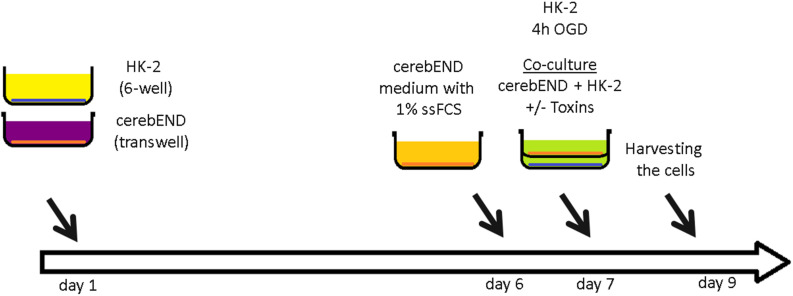
Schematic representation of a co-culture model of proximal tubule kidney cells and brain microvascular endothelial cells. Human proximal tubule kidney cells (HK-2) were grown to confluence in a 6-well plate and treated with oxygen/glucose deprivation (OGD) for 4 h. Brain microvascular endothelial cells (BMEC, cerebEND) were grown to confluence in transwells. The transwells with confluent cerebEND were put together with HK-2 and additionally treated with kidney injury toxins indoxyl sulfate (IS) and indole-3-acetic acid (IAA). The cells were harvested 48 h later.

CerebEND cells were isolated and immortalized as previously described (Silwedel and Forster, 2006; Burek et al., 2012). The cells were grown in Dulbecco’s Modified Eagle’s Medium (DMEM) supplemented with 10% fetal calf serum (FCS) and 1% penicillin/streptomycin in 37°C and 5% CO_2_ on plates coated with 0.5% gelatin. The immortalized human proximal tubule epithelial cells HK-2 (ATCC) were grown in DMEM supplemented with 10% FCS and 1% penicillin/streptomycin on plates coated with 0.5% gelatin. CerebEND cells were grown to confluence for 5 days, followed by differentiation in growth medium with 1% charcoal stripped FCS (without hormone and growth factors) for 24 h. For oxygen/glucose deprivation experiments, confluent HK-2 cells were grown in glucose-free DMEM with 1% FCS for 4 h in 1% O_2_, 5% CO_2_ with or without 1 mM IS and/or 1mM IAA. After 4 h OGD, glucose was added to the growth medium to a final concentration of 4g/l and HK-2 cells were used for co-culture with cerebEND cells. For co-culture, cerebEND cells were seeded in 6-well transwells (0.4 μm pores) and grown as described above (Figure 1). On day 6, the growth medium was replaced with the medium harvested from OGD-treated HK-2 cells supplemented with glucose with or without addition of 1 mM IS and/or 1 mM IAA. The cells were co-cultured for 48 h and then harvested for RNA and protein isolation as described below.

### Real-Time PCR

RNA isolation and real-time PCR analysis were performed as described previously (Burek et al., 2014; Gerhartl et al., 2020). Briefly, total RNA was isolated using the Nucleospin-RNAII Kit (Macherey Nagel) according to the manufacturer’s instructions. We used 1 μg of total RNA for reverse transcription with the High capacity cDNA synthase Kit (Thermo Fisher Scientific). Quantitative PCR was performed using commercially available Taqman^®^ probes (Thermo Fisher Scientific). Calnexin (Canx) was used as endogenous control for the normalization and calculation of the relative expression by the comparative Ct method. Each sample was analyzed in triplicate. The measurements were carried out with the StepOnePlus Real-Time PCR System (Thermo Fisher Scientific).

### Western Blot

Western blot was performed as previously described (Burek et al., 2010; Blecharz et al., 2014). Briefly, the cells were washed with ice-cold PBS and harvested on ice in 50 μl RIPA buffer (50 mM Tris, pH 8; 150 mM NaCl, 0.1% SDS, 0.5% sodium-deoxycholate, 1% NP40) supplemented with proteases and phosphatase inhibitor cocktail (Roche Applied Science). Protein content was quantified using the BCA protein Assay Kit (Thermo Fisher Scientific). Equal amounts of protein (20 μg) were subjected to SDS-PAGE electrophoresis. The proteins were transferred to a Hybond nitrocellulose membrane (Promega), which was blocked with 10% low-fat milk in PBS and incubated overnight at 4 °C with the respective primary antibody in blocking solution against Bcrp (1:1000 Abcam #Ab-24114), Cldn5 (1:200, Thermo Fisher Scientific, #34-1600), Glut-1 (1:200, Millipore #07-1401), Lrp1 (1:1000, Abcam #Ab92544), Mct1 (1:200 Santa Cruz Biotechnology #sc-14917), Mrp4 (1:1000, Enzo Life Science #ALX-801-039-C100), Ocln (1:200, Acris #AP26410PU-N), P-gp (1:200, Enzo Life Sciences #ALX-801-002), TFR (Transferrin Receptor, 1:500, Thermo Fisher Scientific #13-6800). β-actin was used as endogenous control (1: 20 000, Sigma-Aldrich #A3854). Horseradish peroxidase-labeled anti-mouse (Roche Applied Science), anti-rabbit (Cell Signaling Technology), anti-goat and anti-guinea pig (Santa Cruz Biotechnology) IgGs in a dilution of 1:3000 in blocking solution were used as secondary antibodies. The images were taken using an enhanced chemiluminescence detection reagents and FluorChem FC2 Multi-imager II (Alpha Innotech). Densitometric analysis was performed using the Image J software.

### Statistical Analysis

The data are expressed as the mean ± standard deviation. To compare three or more groups, a one-way ANOVA followed by Dunnett’s multiple comparisons test was performed using the GraphPad Prism 7.00 Software. P values less than 0.05 were considered significant.

## Results

### Uremic Toxins and Co-culture With Stressed Kidney Cells Induce the Expression of Efflux Pumps in the BMEC Cell Line CerebEND

IS and IAA are uremic toxins that are upregulated in kidney ischemia/reperfusion injury. IS/IAA were selected for the treatment of brain microvascular endothelial cells (BMEC) to mimic the uremic conditions in the cell culture medium (Yu et al., 2011; Edamatsu et al., 2014). Concentrations of 0.5 mM, 1 mM, 5 mM, and 10 mM of IS and IAA were examined for their toxic effects on BMEC during 24- and 48-h treatment (results not shown). IS and IAA at concentration of 0.5 mM and 1 mM had no toxic effect on BMEC, while higher concentrations were toxic. We selected the non-toxic 1mM concentration of IS and IAA for further experiments. These non-toxic concentrations also had no significant effects on mRNA and protein expression in BMEC (results not shown).

**FIGURE 2:**
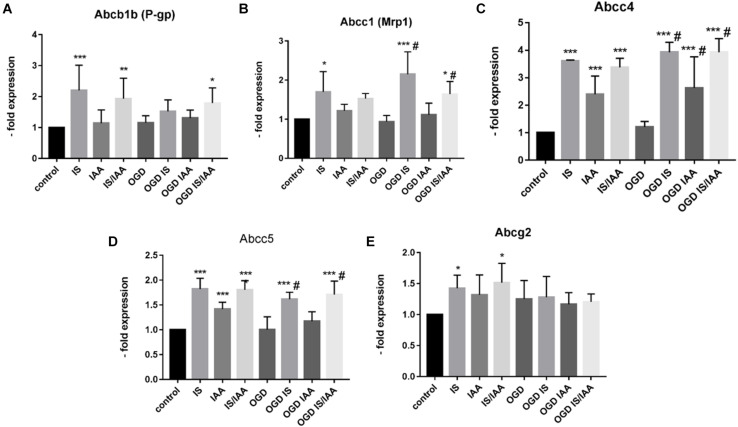
Effects of ischemic HK-2 and uremic toxins on efflux pumps mRNA in brain microvascular endothelial cells. Mouse brain microvascular endothelial cells (BMEC) treated as described in Figure 1 were lysed and used for RNA extraction. The expression of the efflux transporters Abcb1b (P-gp, Mdr1, ATP Binding Cassette Subfamily B Member1) **(A)**, Abcc1 (Mrp1, ATP Binding Cassette Subfamily C Member1) **(B)**, Abcc4 (Mrp4, ATP Binding Cassette Subfamily C Member 4) **(C)**, Abcc5 (Mrp5, ATP Binding Cassette Subfamily C Member 5) **(D)**, and Abcg2 (Bcrp, ATP Binding Cassette Subfamily G Member 2) **(E)** was measured by qPCR. The data are shown as an average of four independent experiments with standard deviations. A one-way ANOVA with Dunnett’s or Sidak’s multiple comparison test was used to compare each treatment to the normoxic cerebEND control without toxin treatment (*p < 0.05, **p < 0.01, ***p < 0.001) or to OGD treatment (#).

**FIGURE 3:**
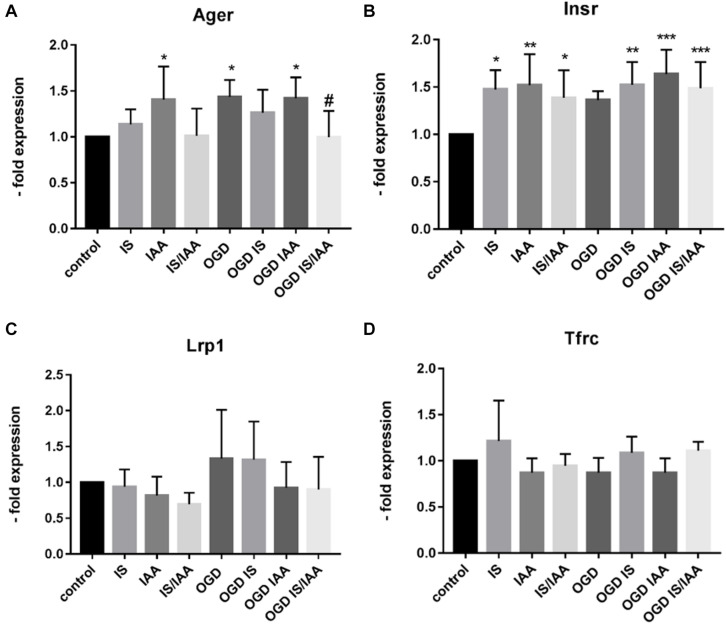
Effects of ischemic HK-2 and uremic toxins on cellular receptor mRNA in brain microvascular endothelial cells. BMECs treated as described in Figure 1 were lysed and used for RNA extraction. The expression of the cellular receptors Ager (Advanced Gylcosylation End-Product Specific Receptor) **(A)**, Insr (Insulin Receptor) **(B)**, Lrp1 (LDL Receptor Related Protein 1) **(C)**, and Tfrc (Transferrin Receptor) **(D)** was measured by qPCR. The data are shown as average of four independent experiments with standard deviations. One-way ANOVA with Dunnett’s or Sidak’s multiple comparison test was used to compare each treatment with the normoxic cerebEND control without toxin treatment (*p < 0.05, **p < 0.01, ***p < 0.001) or to OGD treatment (#).

**FIGURE 4:**
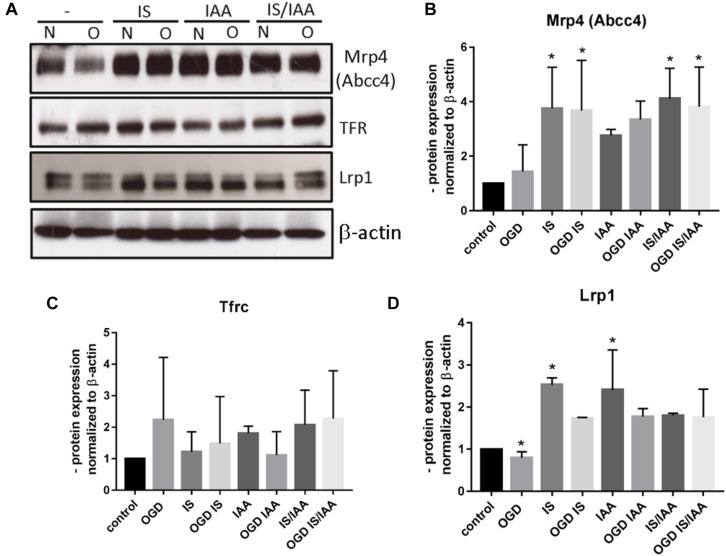
Effects of ischemic HK-2 and uremic toxins on efflux pumps and cellular receptor protein level in brain microvascular endothelial cells. BMECs treated as described in Figure 1 were lysed and used for protein extraction. The protein content of Mrp4 (Abcc4, ATP Binding Cassette Subfamily C Member 4), Tfrc (Transferrin Receptor), and Lrp1 (LDL Receptor Related Protein 1) was measured by Western blot **(A)**. The protein content was normalized to β-actin and to the untreated control. The densitometric analysis is shown as an average of four independent experiments with standard deviations **(B–D)**. One-way ANOVA with Dunnett’s or Sidak’s multiple comparison test was used to compare each treatment to the untreated control. *p < 0.05.

There may be an undefined mixture of toxins in the blood during kidney ischemia/reperfusion injury (Lu et al., 2015). In order to increase the number of kidney-derived factors in the cell culture medium, we co-cultured the human proximal tubule kidney cells HK-2 with BMEC as shown schematically in Figure 1. HK-2 cells were stressed by 4-h oxygen-glucose deprivation (OGD), as previously established (Kleinschnitz et al., 2011; Burek et al., 2019; Ittner et al., 2020) and were then cultured along with BMEC (Forster et al., 2005; Silwedel and Forster, 2006; Burek et al., 2012; Helms et al., 2016). OGD shows similar effects on kidney cells as AKI (Sauvant et al., 2009). HK-2 were incubated in glucose-free medium under 1% O_2_. After 4 h, glucose was added to a final concentration of 4 g/ml. The HK-2-secreted factors during 4-h OGD period were preserved in medium. After that, the confluent BMEC seeded in transwells were placed to HK-2 wells and received a HK-2 conditioned medium. In addition, uremic toxins were added to selected transwells (Figure 1). A co-culture with HK-2 without OGD treatment was used as a control. BMEC were harvested 48 h later and were subjected to qPCR and Western blot. First, we analyzed the main efflux pumps that are expressed on the BBB. At the mRNA level, all efflux pumps examined were elevated due to treatment with uremic toxins and co-culture with either normoxic or OGD-treated HK-2 cells (Figures 2A–E). Interestingly, OGD-stressed HK-2 alone did not induced expression of efflux pumps in BMECs probably because of the recovery of the cells during the 48 h reoxygenation. The strongest effects were observed on Abcc4 and Abcc5 expression (Figures 2C,D) with 4- and 2-fold increased expression after IS, IAA or combination of both in either normoxic or OGD-treated HK-2 co-culture. The genes coding for proteins involved in the receptor-mediated transport at the BBB showed a moderate increase in the case of Ager and Insulin receptor (Insr) (Figures 3A,B). No significant changes in the Lrp1 and transferrin receptor (Tfrc) mRNA were found. We then examined the protein levels of Mrp4, Lrp1 and Tfr (Figure 4). Similarly to Abcc4 mRNA, the Mrp4 protein in BMEC treated with either IS or IS/IAA in co-culture with normoxic or OGD-treated HK-2 cells was significantly increased (Figures 4A,B). Lrp1 protein level was decreased due to OGD and increased in IS- and IAA-treated BMEC in co-culture with normoxic HK-2 (Figure 4D). Tfr protein level was not significantly changed under any condition (Figure 4C).

### Uremic Toxins and Co-culture With OGD-Stressed Kidney Cells Have no Effects on the Expression of Solute Carrier Transporters

**FIGURE 5:**
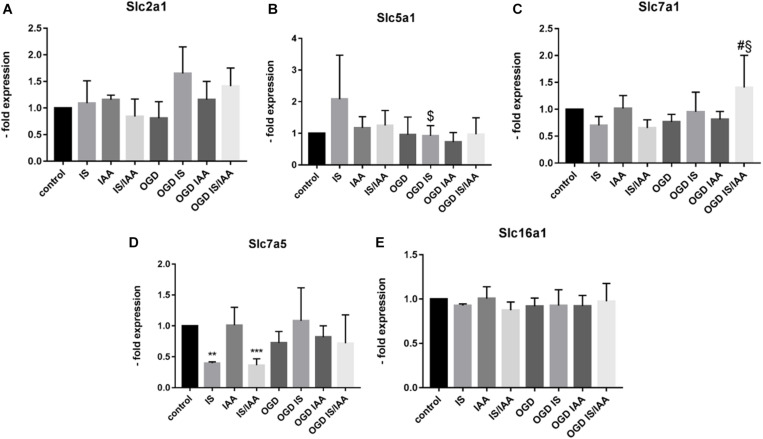
Effects of ischemic HK-2 and uremic toxins on solute carrier transporter mRNA in brain microvascular endothelial cells. BMECs treated as described in Figure 1 caption were lysed and used for RNA extraction. The expression of solute carrier transporter mRNA Slc2a1 (Glut-1, Solute Carrier Family 2 Member 1) **(A)**, Slc5a1 (Sglt1, Solute Carrier Family 5 Member 1) **(B)**, Slc7a1 (Cat1, Solute Carrier Family 7 Member 1) **(C)**, and Slc7a5 (Lat1, Solute Carrier Family 7 Member 5) **(D)**, and Slc16a1 (Mct1, Solute Carrier Family 16 Member 1) **(E)** was measured by qPCR. The data are shown as an average of four independent experiments with standard deviations. One-way ANOVA with Dunnett’s or Sidak’s multiple comparison test was used to compare each treatment to the untreated cerebEND control (**p < 0.01, ***p < 0.001) or to OGD treatment (#), $: statistically significant versus IS, §: statistically significant versus IS/IAA.

**FIGURE 6:**
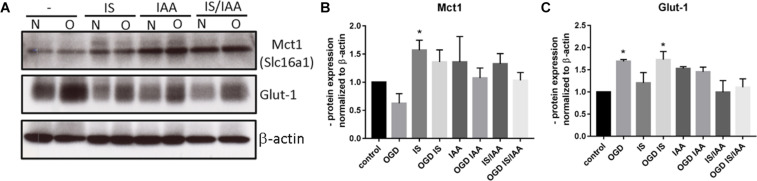
Effects of ischemic HK-2 and uremic toxins on the protein level of solute carrier transporters in brain microvascular endothelial cells. BMECs treated as described in Figure 1 were lysed and used for protein extraction. The protein content of Mct1 (Slc16a1, Solute Carrier Family 16 Member 1) and Glut-1 (Slc2a1, Solute Carrier Family 2 Member 1) was measured by Western blot **(A)**. The protein content was normalized to β-actin and the untreated control. The densitometric analysis is shown as an average of four independent experiments with standard deviations **(B,C)**. One-way ANOVA with Dunnett’s multiple comparison test was used to compare each treatment to the control. *p < 0.05.

**FIGURE 7:**
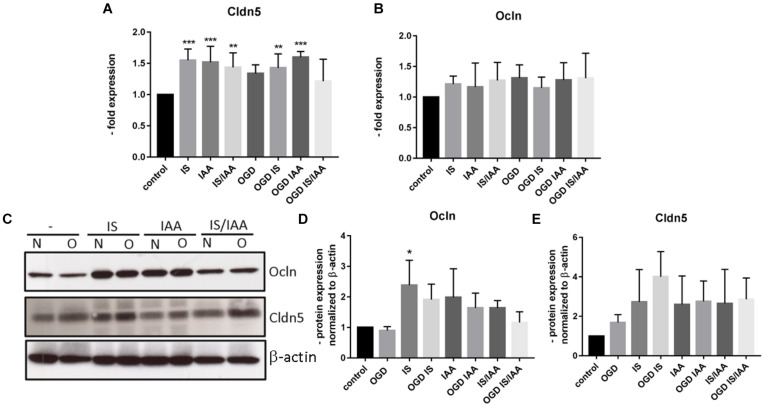
Effects of ischemic HK-2 and uremic toxins on the mRNA and protein levels of tight junction proteins in brain microvascular endothelial cells. BMECs treated as described in Figure 1 were lysed and used for RNA and protein extraction. Expression of tight junction protein Cldn5 (claudin-5) **(A)** and Ocln (occludin) **(B)** mRNA was measured by qPCR. The protein content was quantified by Western blot **(C–E)**. β-actin was used as an endogenous control. The data are shown as an average of four independent experiments with standard deviations. One-way ANOVA with Dunnett’s multiple comparison test was used to compare each treatment to the control. *p < 0.05, **p < 0.01, ***p < 0.001.

Solute carrier transporters (Slc) are strongly expressed at the BBB and are responsible for supplying the brain with nutrients such as glucose, amino acids or ions (Nalecz, 2017). We examined selected Slc genes, such as Slc2a1 (Glut1, glucose transporter), Slc5a1 (Sglt-1, Na + /Glucose Cotransporter), Slc7a1 (Cat1, Cationic Amino Acid Transporter), Slc7a5 (Lat1, Large Neutral Amino Acids Transporter) and Slc16a1 (Mct 1, Monocarboxylate Transporter) (Figure 5). Slc7a5 showed reduced expression after treatment with IS and IS/IAA in co-culture with normoxic HK-2 cells. The effects of IS on Slc5a1 were lower when combined with OGD, while OGD potentiated the effects of IS/IAA on Slc7a1. All other Slc mRNAs showed no changes under all experimental conditions. At the protein level, Mct1 (Slc16a1) was induced by IS treatment in co-culture with normoxic HK-2 (Figure 6). Under other conditions, the protein content was higher than in the control, but not statistically significant (Figure 6). Glut-1 (Slc2a1) protein levels were significantly increased in co-culture with OGD-treated HK2 cells with or without IS (Figure 6). We next examined the mRNA (Figures 7A,B) and protein expression (Figures 7C–E) of the tight junction proteins claudin-5 and occludin. The mRNA levels of Cldn5 were increased in all experimental arrangements compared to the control (Figure 7A), but these differences were not visible at the protein level (Figures 7C,D). Occludin expression was increased due to IS treatment, but remained unchanged at both mRNA and protein level (Figures 7B–D) under different conditions. Overall, claudin-5 and occludin showed increased mRNA and protein levels, without reaching the significance in most treatments.

**FIGURE 8:**
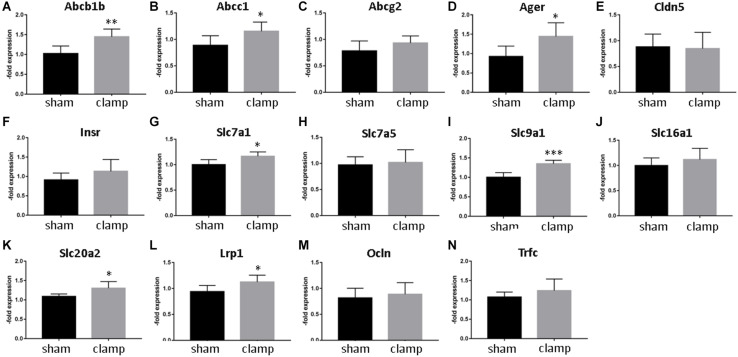
Expression of selected transporters and tight junctions proteins in brain capillaries from mice exposed to bilateral renal ischemia/reperfusion injury on mRNA level. Brain capillaries were isolated from frozen brains of mice exposed to bilateral renal ischemia/reperfusion injury followed by RNA isolation and qPCR. Expression of Abcb1b (P-gp, Mdr1, ATP Binding Cassette Subfamily B Member1) **(A)**, Abcc1 (Mrp1, ATP Binding Cassette Subfamily C Member1) **(B)**, Abcg2 (Bcrp, ATP Binding Cassette Subfamily G Member 2) **(C)**, Ager (Advanced Gylcosylation End-Product Specific Receptor) **(D)**, Cldn5 (Claudin-5) **(E)**, Insr (Insulin Receptor) **(F)**, Slc7a1 (Cat1, Solute Carrier Family 7 Member 1) **(G)**, Slc7a5 (Lat1, Solute Carrier Family 7 Member 5) **(H)**, Slc9a1 (Nhe1, Solute Carrier Family 9 Member a1) **(I)** Slc16a1 (Mct1, Solute Carrier Family 16 Member 1) **(J)**, Slc20a2 (Pit2, Solute Carrier Family 20 Member 2) **(K)**, Lrp1 (LDL Receptor Related Protein 1) **(L)**, Ocln (Occludin) **(M)**, and Trfc (Transferrin Receptor) **(N)** was calculated by relative quantification method and is shown as average of *n* = 3 with standard deviations. *p < 0.05, **p < 0.01, ***p < 0.001.

**FIGURE 9:**
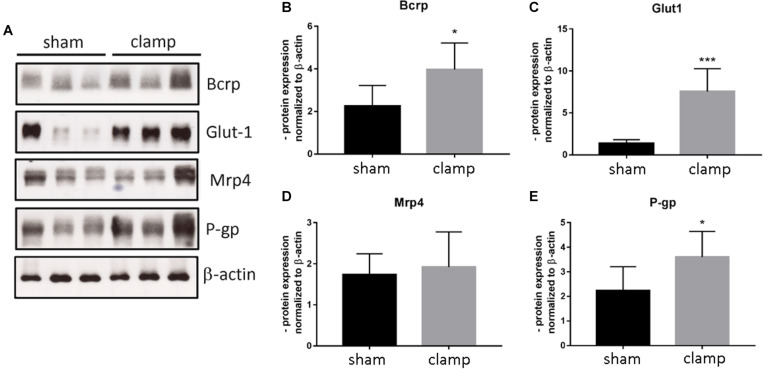
Expression of selected transporters and tight junction proteins in brain capillaries of mice exposed to bilateral renal ischemia/reperfusion injury at the protein level. Brain capillaries were isolated from frozen brains of mice exposed to bilateral renal ischemia/reperfusion injury followed by protein extraction and Western blot **(A)**. The protein content of Bcrp (Abcg2, ATP Binding Cassette Subfamily G Member 2) **(B)**, Glut1 (Slc2a1, Solute Carrier Family 2 Member 1) **(C)**, Mrp4 (Abcc4, ATP Binding Cassette Subfamily C Member 4) **(D)**, and P-gp (Mdr1, Abcb1b ATP Binding Cassette Subfamily B Member1) **(E)** was normalized to β-actin and is shown as average of *n* = 3 with standard deviations. *p < 0.05, ***p < 0.001.

To validate our *in vitro* results, we used isolated capillaries from frozen brains from mice that had undergone bilateral renal ischemia (30 minutes)/reperfusion (24 h) injury (Schneider R, unpublished) for mRNA and protein analysis (Figures 8, 9). The capillaries of control mice (sham) and mice with renal ischemia/reperfusion injury (clamp) were examined for the expression of transporters, receptors and tight junction proteins. Among the efflux pumps, Abcb1b and Abcc1 were significantly increased in the clamp (Figures 8A,B). Abcg2 mRNA showed no changes (Figure 8C). The mRNA expression of solute carrier transporters Slc7a1, Slc9a1 and Slc20a2 was significantly increased in brain capillaries of uremic mice (Figures 8G,I,K). The mRNA of Slc7a5 and Slc16a1 remained unchanged (Figures 8H,J). Among the cellular receptors, the Ager and Lrp1 mRNAs were significantly increased in the clamp (Figures 8D,L) while no differences were seen in Insr and Trfc mRNA expression (Figures 8F,N). No changes in Cldn5 and Ocln mRNA expression were found (Figures 8E,M). We examined the protein expression of selected efflux and solute carrier transporters in capillaries of sham and clamp mice (Figure 9). Bcrp (Abcg2), P-gp (Abcb1) and Glut-1 (Slc2a1) (Figures 9A–C,E) were significantly increased at the protein level in brain capillaries of clamp mice. The Mrp4 (Abcc4) protein was not changed (Figures 9A,D).

In summary, we show that uremic conditions affect the BMEC *in vivo* and *in vitro* by altering the protein and mRNA expression of relevant efflux and influx transporters at the BBB.

## Discussion

Kidney failure not only leads to kidney damage, but can also have systemic effects on distant organs. Experiments with uremic mice showed cerebral inflammation (Liu et al., 2008) and in some cases cerebral edema (Kim et al., 2016). In this study, we used BMEC co-culture with kidney cells and treatment with the uremic toxins IS and IAA as *in vitro* model for kidney-brain crosstalk in the kidney ischemia/reperfusion injury model. We analyzed the effects of uremic toxins on BMEC and compared our results with the brain capillaries of mice exposed to renal ischemia/reperfusion injury. We selected the uremic toxins IS and IAA for treatment because they have previously been shown to affect endothelial cells (Gondouin et al., 2013). The limitation of our study is that we only used two selected uremic toxins. *In vivo*, multiple toxins accumulate in the blood during AKI due to the decreased GFR of dysfunctional kidneys. Most toxins are bound to albumin in the blood, so their total toxin concentration is unknown (Duranton et al., 2012). We selected a sub-toxic concentration of IA and IAA, which is much higher than that reported in patients (Vanholder et al., 2003). Co-culture with OGD-stressed kidney cells increased the amount of toxins and other kidney factors in the cell culture medium, but the effect of toxin accumulation over time could not be achieved *in vitro*. The neurological symptoms show only a low correlation with the uremic toxin concentrations in patients with kidney failure and mostly occur in the acute phase of AKI (Burn and Bates, 1998). The experimental times chosen for the experiments correspond to the acute phase of AKI. The *in vitro* and *in vivo* setting with isolated brain capillaries enables the molecular analysis of BMEC during AKI.

The level of tight junction proteins claudin-5 and occludin expression correlates with the tightness of the BBB (Balda et al., 1996; Forster et al., 2005; Burek et al., 2010; Kaiser et al., 2018). In our kidney-brain crosstalk *in vitro* model and in the brain capillaries of mice with kidney ischemia/reperfusion injury, we could not find any major differences in tight junction protein expression. Only claudin-5 was significantly increased on mRNA level in BMEC. These effects were not visible at the protein level during the selected experimental times.

The glucose transporter showed an increased expression both in stressed cells and in mice capillaries. This indicates the brain endothelial cells are actively responding to toxins and ischemia/reperfusion injury factors and are trying to compensate for the changes in reduced metabolic activity (Burn and Bates, 1998) by increasing glucose transport and supporting the brain with nutrients. This increased Glut-1 expression is also in line with the hyperglycemia observed during AKI (Husi and Human, 2015). However, all other solute carrier transporter showed no significant changes *in vitro*. *In vivo*, an increased expression of solute carrier transporters was observed at the mRNA-level. Slc20a2 (sodium-dependent phosphate transporter), Slc9a1, and Slc7a1 showed a significantly increased mRNA-expression. Slc9a1 (Nhe1), a sodium-hydrogen antiporter 1, is involved in the volume and pH-regulation of vertebrate cells, and its increase may be one of the protective mechanisms of BMECs against uremic factors. Slc7a1 and Slc7a5 (Cat1, cationic amino acid transporter 1 and Lat1, large neutral amino acids transporter small subunit 1) were increased in the brain capillaries after kidney ischemia/reperfusion injury. Slc7a5 and Slc16a1 showed the same tendency. Slc16a1 (Mct1, monocarboxylate transporter 1) is the main regulator of the activation of the Hypoxia activated factor (Hif-1) by lactate in endothelial cells (Sonveaux et al., 2012). Mct1 was also induced *in vitro* by treating BMECs with IS. At 48 h of *in vitro* treatment, most changes in expression were observed for the efflux pumps and not for solute carrier transporters. The cells try to pump out the toxins and the transport of only the most relevant nutrient glucose is increased. The reason for the low effects of co-culture with OGD-treated HK-2 cells on BMECs is the time chosen for sampling. The HK-2 cells were subjected to 4-h OGD and then reoxygenated for 48 h while co-cultured with BMECs with or without toxin treatment. The cells recovered and the demonstrated OGD effects on BMECs were low. However, the effects on a hypoxia/aglycemia marker Glut-1 were still visible at the protein level, which confirmed the correct experimental setting. The recovery during reoxygenation could also be observed in OGD-treated BMECs as published by us and others (Salvador et al., 2015; Tornabene et al., 2019).

We were able to show the effects of the two uremic toxins IS and IAA on BMECs. Our results further confirm the impact of IS and IAA on neurological symptoms of AKI (Iwata et al., 2007). IS has been shown to change drug delivery at the BBB (Ohtsuki et al., 2002). In our hands, IS and IAA also induced multiple changes in the gene and protein expression of drug transporters at the BBB. It will be interesting to use serum from AKI patients to treat the BMEC *in vitro*. The human BMEC would be the best choice for these studies.

Insr mRNA showed a significant increase in BMECs due to the treatment with IS and IAA under normoxic or OGD conditions. The same tendency was found for Insr in capillaries from mice with kidney ischemia/reperfusion injury. The increase in insulin signaling corresponds to the literature, in which it was shown, that inhibition of insulin signaling delays the onset of kidney failure (Ising et al., 2015).

The observed alterations at the BBB might play a role in the etiology of reported short- and long-term effects of AKI on brain function. Short-term effects are uremic encephalopathy while long-term effects are associated with a risk for developing stroke and dementia (Grams and Rabb, 2012; Guerra et al., 2012; Wu et al., 2014; Tsai et al., 2017). Studies with uremic mice showed that AKI leads to inflammation and functional changes in the brain (Liu et al., 2008). The authors of the study focused on the hippocampus, but effects on amygdala, insular and cingulate gyrus could be also possible as a background for the functional changes leading specifically to dementia. Moreover, an autonomic imbalance was suggested for the pathophysiology in CKD or AKI (Tanaka and Okusa, 2020), so that an impact of uremic toxin exposure on the autonomic areas of the brain could be associated with this relationship.

## Data Availability Statement

The original contributions presented in the study are included in the article/supplementary material, further inquiries can be directed to the corresponding authors.

## Ethics Statement

All animal experiments were approved by the local animal care committee (Animal Care and Use Committee, Government of Lower Franconia, Wuerzburg, Germany).

## Author Contributions

MB, SB, ES, and MN performed and analyzed the experiments and drafted the manuscript. KM-E and RS provided frozen brains from uremic mice. MB, NR, RS, and CYF designed the study. All authors read and approved the final version of the manuscript.

## Conflict of Interest

The authors declare that the research was conducted in the absence of any commercial or financial relationships that could be construed as a potential conflict of interest.
